# Memory shapes visual search strategies in large-scale environments

**DOI:** 10.1038/s41598-018-22731-w

**Published:** 2018-03-12

**Authors:** Chia-Ling Li, M. Pilar Aivar, Matthew H. Tong, Mary M. Hayhoe

**Affiliations:** 1 Center for Perceptual Systems, The University of Texas at Austin, Austin, Texas USA; 2 Facultad de Psicología, Universidad Autónoma de Madrid, Madrid, Spain; 3 IBM Research, Austin, Texas USA

## Abstract

Search is a central visual function. Most of what is known about search derives from experiments where subjects view 2D displays on computer monitors. In the natural world, however, search involves movement of the body in large-scale spatial contexts, and it is unclear how this might affect search strategies. In this experiment, we explore the nature of memory representations developed when searching in an immersive virtual environment. By manipulating target location, we demonstrate that search depends on episodic spatial memory as well as learnt spatial priors. Subjects rapidly learned the large-scale structure of the space, with shorter paths and less head rotation to find targets. These results suggest that spatial memory of the global structure allows a search strategy that involves efficient attention allocation based on the relevance of scene regions. Thus spatial memory may allow less energetically costly search strategies.

## Introduction

In natural behavior, visual information is actively sampled from the environment by a sequence of gaze changes. These gaze changes reflect shifts of attention, and are frequently the result of a visual search operation that specifies a region of the peripheral retina as the target of the next eye movement. Locations in scenes are selected as gaze targets in order to direct the fovea to a region where new information can be gathered for ongoing visual or cognitive operations. A large body of work in visual search has revealed many of the factors that influence the search efficiency, such as the stimulus features of the image, top down guidance, and scene semantics^1^. However, most of this work has been done using 2D displays on computer monitors, and only a relatively small number of studies have examined visual search in the natural world^2–4^. This is an important issue because the nature of the stimulus in standard experimental paradigms differs considerably from that in everyday experience^3,5–7^. For example, experimental paradigms typically entail brief exposures to a large number of different images, whereas in natural settings humans are immersed in a relatively small number of environments for longer durations. This provides viewers with the opportunity to develop memory representations of particular environments, and to use such representations to guide search, rather than interrogating the visual image.

In addition to the greater opportunity to build memory representations in normal experience, the involvement of body movements may influence the development of such representations. For example, when moving around a room it is necessary to store information about the spatial layout in order to be able to orient to regions outside the field of view. Land *et al*.^8^ noted instances when subjects made large gaze shifts to locations outside the field of view that involved eye, head, and body movements, and were remarkably accurate. Another aspect of 3D environments is that movement is self-initiated and accompanied by proprioceptive and vestibular feedback that allow the development of abstract spatial memory representations^9^. These components of active behavior are absent in experiments that attempt to understand scene learning and visual search using 2D stimuli. Jiang *et al*.^3^ investigated visual search in an outdoor environment and found that the ability to move the body during search allowed subjects to code targets in both egocentric and allocentric frames, whereas coding in laboratory environments was primarily egocentric. They also demonstrated that subjects learnt statistically likely regions for the target location and used this to speed up search. Thus the structure of memory representations and strategies for search are likely influenced by experience in immersive environments.

Another factor to consider in understanding the role of memory is the cost of the movements when exploring in large spaces, which is presumably tied to the pervasive effects of the neural reward machinery^10–12^. For example, in locomotion, subjects choose a preferred gait that reflects the energetic minimum determined by their body dynamics^13^. Other experiments have demonstrated that subjects store information in spatial memory if a large head movement is needed to update the information, presumably because head movements are energetically costly^14–17^. In a real world search task, Foulsham *et al*.^18^ found that 60–80% of the search time was taken up by head movements, making minimization of such movements a significant factor. Visual search typically occurs in complex, multi-room environments, and involves planning and coordination of movements of the eyes, head and body through space^18–20^. Use of spatial memory representations should therefore allow more efficient planning of the path through the space.

Experiments on the role of memory in visual search have revealed a complex picture. In 2D experiments, subjects often do not use memory when searching is easy without accessing memory^21^. On the other hand, the semantic structure of the scene, learnt from past experience, is an important factor in allowing more efficient search in both 2D and 3D environments^22,23^. In 2D environments, the locations of objects that have been searched for previously are remembered, but objects fixated incidentally are not^24^. Previously we examined visual search in immersive virtual apartments, and found similar results to Võ and Wolfe, where memory was good for task relevant objects, but not for other objects in the room^25,26^. In the present experiment we explore further how memory is used to guide search in immersive environments. Our previous results suggested that much of the advantage of experience might come from learning the spatial layout of the apartment and remembering the correct room to explore. Previous work also suggested that spatial memory may be organized hierarchically, where object location is represented relative to a sub-region of the environment, and the sub-region is represented relative to the larger scale environment^27,28^. In the present experiment we explore representations at different spatial scales are used to aid search. Do subjects remember the exact coordinates of the target or a less precise location, such as the side of the room the target is on? If memory is at a coarse scale such as which room, or which side of the room, or which surface, then it may be faster to use visual cues rather than an imprecise memory, especially when the targets are relatively easy to locate and the target is within the field of view.

**Figure 1:**
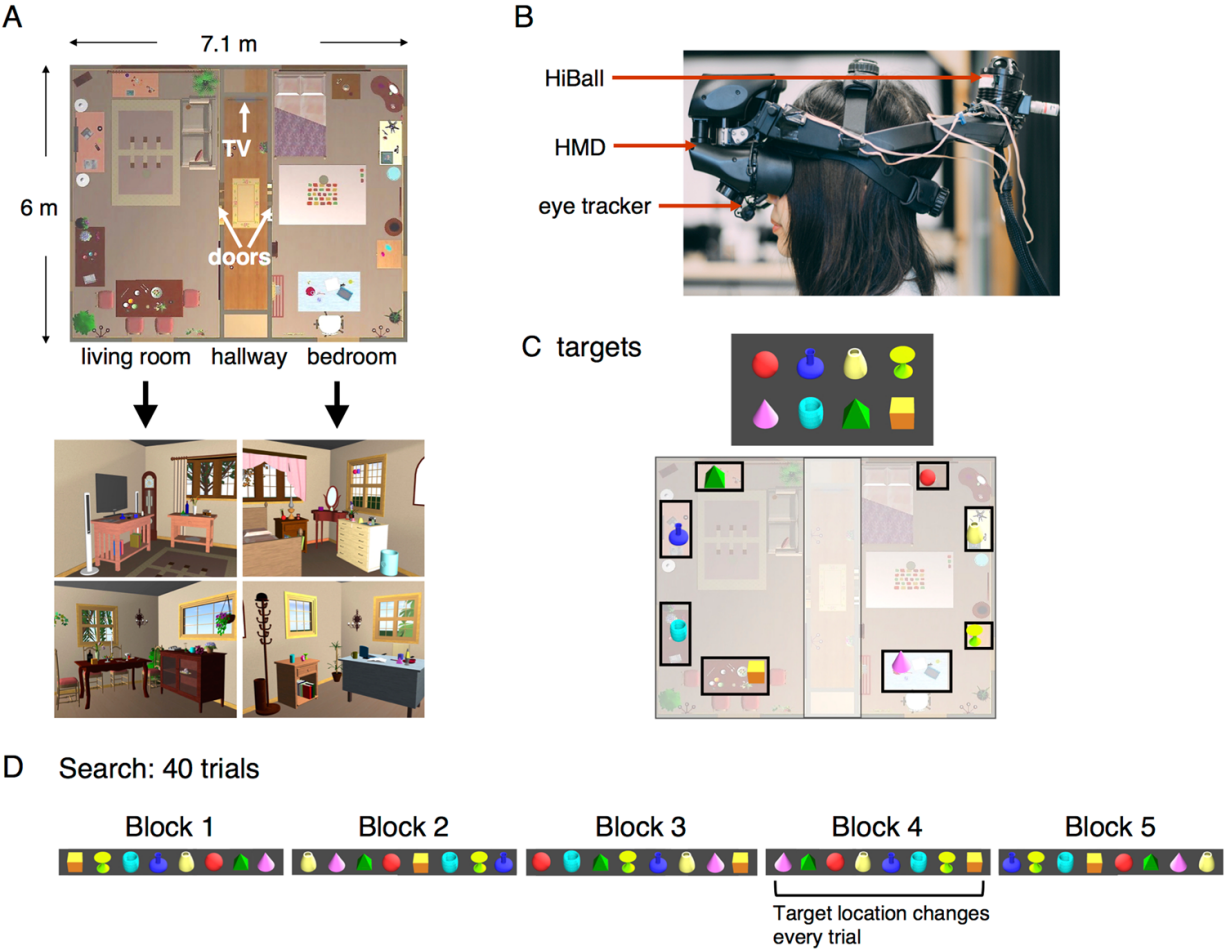
Experimental setup. (**A**) Virtual apartment. Top: bird’s eye view of the environment. On each trial, the target was shown on the TV at the end of the hallway when subjects approached it. Subjects then searched for the target in the two rooms. Bottom: views of the living room (left) and bedroom (right). (**B**) A subject wearing the head-mounted display (HMD), which was equipped with an Arrington eye tracker and a HiBall head position tracker. (**C**) Top: target objects. Bottom: illustration of the placements of the targets on eight surfaces across the two rooms. Note that the sizes of the targets are enlarged for illustrative purposes. (**D**) An example trial sequence of five search blocks. Locations of targets are constant in Block 1–3 and Block 5. In Block 4, all target objects were shuffled to a different location in either the same room or the other room in each trial.

To probe how memory was used, we employed the same virtual reality apartment as in Li *et al*.^25^. Subjects searched for targets on three occasions. We then randomly changed the target locations and asked how this affected search (Fig. 1). If subjects use a visually based search strategy once they are in the correct room, this manipulation would have little effect. On the other hand, if the specific target location relative to the spatial layout of the apartment is remembered, subjects may orient their gaze to the previous target location despite the fact that it is no longer there. We found that subjects frequently fixated the previous target location despite its absence, indicating that, in these instances, search was driven by memory of spatial location. Additionally, we found that attention increasingly narrowed down to relevant parts of the scene at different spatial scales. Finally, we examined whether the cost of movement was a factor in determining the usage of memory. We show that the total distance traveled and the extent of head movements decreased sharply with experience. Thus visual search in natural environments is strongly influenced by spatial memory and this may reflect the constraints imposed by moving the body in space.

## Results

### Effect of changing target location on search

**Figure 2:**
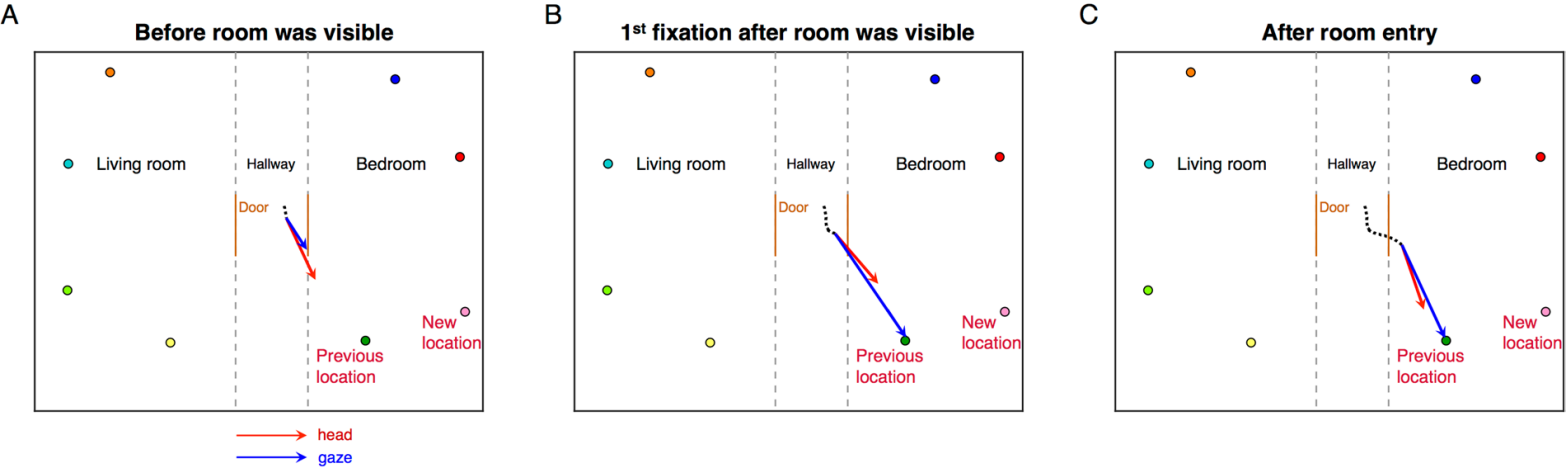
Simplified bird’s eye view of the environment and the head and gaze direction on a trial in Block 4, when a subject searched for a pink target that was displaced to a nearby surface. A green object occupied the previous location of the pink target. The other colored dots are other target objects. The black dots on the hallway show the head position history. The red arrow indicates the direction of head and the blue arrow indicates the direction of gaze. (**A**) Before the room was visible, the subject was pointing her head and gaze to the previous target location. (**B**) The first fixation after the room was visible was made to the previous location of the target. (**C**) About 1 sec later, the subject entered the room, but was still fixating the same, now wrong, location.

We first examined the consequences of moving the targets in the 4^th^ block of trials. An example of gaze and head direction at different points in time during a search trial from one subject is shown in Fig. 2. A movie showing the sequence of gaze and head direction change can be found at https://youtu.be/hgxAo1vOT7w. The subject was searching for a pink cone, which was moved to the new location indicated in the figure. The red and blue arrows in the figure indicate head and eye direction, respectively. Figure 2A shows that the subject was pointing her gaze and head toward the target even before seeing what was inside the room. In Fig. 2B once the room became visible, the subject maintained gaze on the old target location and continued fixating the new object there while approaching it during the next second, before fixating the actual new target location. This behavior suggests that the old target location was stored in spatial memory relative to the coordinates of the apartment and that the fixation was programmed solely on the basis of that memory. We analyze the head movements in more detail below.

Across 8 shuffling trials we found that subjects fixated the previous location of the target on 58.3% of the trials. Of all the fixations made in the room where the target used to reside, 57.9% were made to its previous location. This results in an average of 0.9 fixations to the previous location of the target. Once in the correct room, 30.6% of first fixations and 44.4% of the first two fixations were made to the previous target location. These results suggest that subjects have memory representations of the target locations and are using them to guide gaze on many of the trials. Average fixation duration on the old location was compared with average fixation duration to other locations in the same room, but no differences were found (paired t-test: *t*(8) = 0.94, *p* = 0.38, Cohen’s *d* = 0.31).

**Figure 3:**
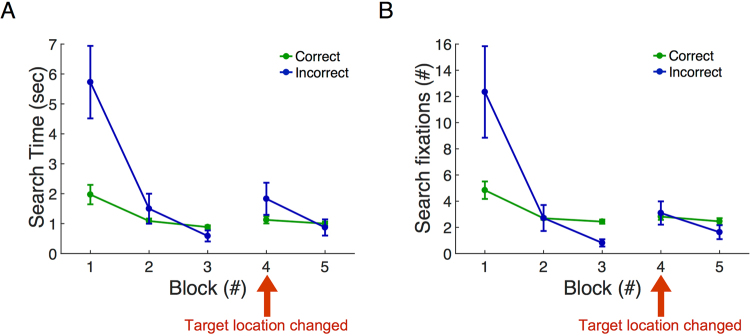
General search performance. (**A**) Search time and (**B**) search fixation counts across search blocks (N = 9). Data represent mean ± SEM across subjects.

Figure 3 shows search time and number of fixations as a function of search block. Each block consists of 8 search trials with a different target on each trial. In repeated blocks the same set of 8 targets were searched for again, in a different random order. Search time and fixations in the correct room (containing the target) are separated from those in the incorrect room (target absent). Note that there were initially large numbers of fixations and long search times in the incorrect room, both of which declined rapidly on the 2^nd^ and 3^rd^ search block (time: Welch’s *F*(2, 12.14) = 9.4, *p* = 0.003, *η*^2^ = 0.52; fixations: Welch’s *F*(2, 11.5) = 6.6, *p* = 0.012, *η*^2^ = 0.42). In the correct room there were fewer fixations and more rapid search initially, which also declined in the first three blocks (time: Welch’s *F*(2, 14) = 6.48, *p* = 0.01, *η*^2^ = 0.42; fixations: Welch’s *F*(2, 14) = 6.06, *p* = 0.013, *η*^2^ = 0.47). In Block 4, when target locations changed, there was a small (but non-significant) increase in fixations and search time in the incorrect room, but essentially no change in fixations in the correct room (Games-Howell test for Block 3 vs. Block 4, time/correct room: *p* = 0.42, time/incorrect room: *p* = 0.26; fixation/correct room: *p* = 0.7; fixation/incorrect room: *p* = 0.18). Thus, as long as subjects choose the correct room by chance on the 4^th^ block, they found the target rapidly even though it was in a new location. This means that subjects also learned general characteristics of the search space that allowed rapid search. In Block 5, when the target locations reverted to their original configuration, search time and number of fixations were similar to those in Block 3. There was a small but non-significant decrease in the number of fixations in the incorrect room (post-hoc test results for Block 3 vs. Block 5, time/correct room: *p* = 0.72, time/incorrect room: *p* = 0.9; fixation/correct room: *p* = 1; fixation/incorrect room: *p* = 0.66). This is most likely accounted for by the reduced probability of going into the incorrect room once memory representations could be used again to guide search.

### Attention and memory structure

**Figure 4:**
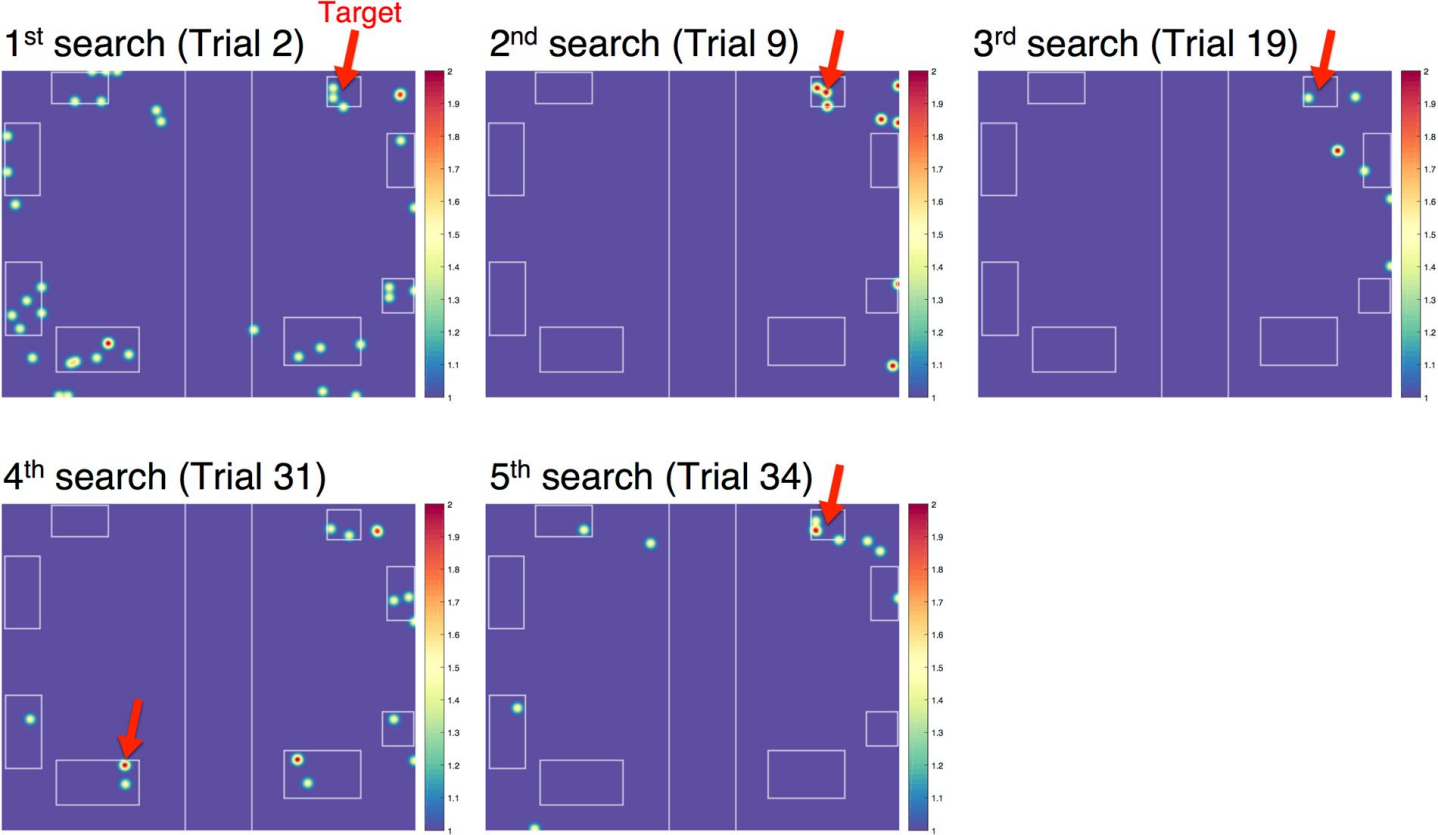
Heat maps showing distribution of fixations at five trials in which a subject searched for a red sphere. The red arrow marks the location of the target. Note the change in target location on the 4^th^ search.

In this section we explore what subjects learned about the space, given the small impact on search performance from changing target location. Figure 4 plots heat maps of gaze distribution on different trials of one subject searching for one specific target, and illustrates how fixations change over search blocks. In the first search block there were numerous fixations in the incorrect room, and a number of fixations on regions other than the four surfaces that contain targets. In the second and third blocks there were fewer fixations overall, and fewer fixations on non-surface areas. When the target was moved to the other room in Block 4, almost all fixations fell on surfaces, even in the incorrect room. When the target returned to its previous location in Block 5, the regions fixated in the correct room were similar to 2^nd^ and 3^rd^ searches.

To examine this further, we tested whether subjects learn to attend to relevant regions and whether the space is represented in a coarse-to-fine manner. To find a target, subjects must first choose the correct room and direct their heads towards the correct side of the room. Within the room there are four surfaces that contain targets, one of which will be correct on the current trial. We explored gaze distribution at these different spatial scales in the first three search blocks.

**Figure 5:**
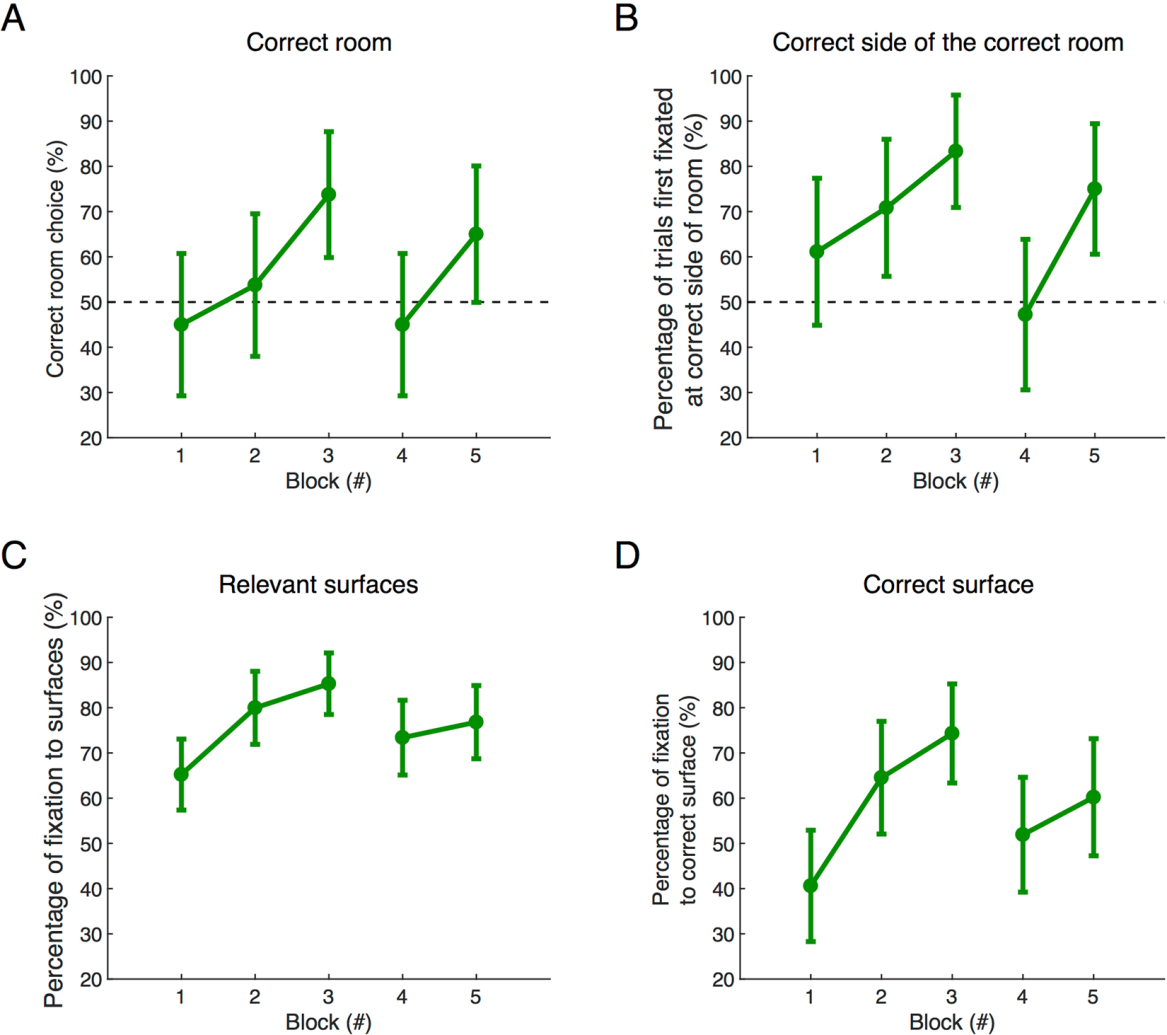
Changes in gaze distribution at different spatial scales over blocks. (**A**) Percentage of trials in which the first room entered was the correct one (N = 19). (**B**) Percentage of trials in which the first fixation was made to the correct side of the room (correct room only, N = 9). (**C**) Percentage of fixations made to the eight relevant surfaces in either room (N = 9). (**D**) Percentage of fixations to the correct surface in the room (N = 9). Data represent mean ± SEM.

First we assessed the percentage of trials in which subjects chose the correct room. Figure 5A shows that by the third block subjects chose the correct room 73.8% of the time, increasing from 45% in Block 1. This increase in accuracy in the first three blocks was significant (*F*(2, 56) = 6.92, *p* = 0.002, *η*^2^ = 0.2). Figure 5B shows the proportion of trials in which initial fixations, once subjects were in the correct room, were on the same side of the room that the target was on. Figure 5C shows the proportion of fixations that were on relevant surfaces, in which other targets were located. Figure 5D shows the proportion of fixations on the correct surface, where the target of the current trial is located. Across the first three blocks there were significant improvements in: (i) the percentage of trials in which subjects first fixated the correct side of the room after it became visible (*F*(2, 23) = 7.07, *p* = 0.004, *η*^2^ = 0.4), (ii) the percentage of trials in which participants fixated relevant surfaces in both the correct and the incorrect room (*F*(2, 214) = 14.9, *p* = 0, *η*^2^ = 0.12), (iii) the percentage of trials in which the correct surface was fixated (*F*(2, 214) = 16.82, *p* = 0, *η*^2^ = 0.14). Thus subjects quickly learned the relevant regions of the space at different scales. Once gaze landed on the correct surface, around half of the time it was on the target. Changing target location in Block 4 also came with the cost of decreasing accuracy. This was true for room choice (*p* = 0.02), side of the room first fixated (*p* < 0.001), fixations to relevant surfaces (*p* = 0.022), and percentage of fixations to correct surfaces (*p* = 0.003).

A notable feature of the change in allocation of attention was the rapid reduction of fixations that occurred, even when subjects went into the incorrect room. Since subjects appeared to rapidly learn to look at surfaces, we analyzed if the faster exclusion of the incorrect room could result from restricting search to surfaces. Fixation count to non-surface areas dropped quickly by 6.28 fixations after Block 1, which is consistent with the rapid improvement in search performance. Fixation count to non-surface areas in the incorrect room also dropped significantly in the first three blocks (Welch’s *F*(2, 96.3) = 10.28, *p* = 0, *η*^2^ = 0.076). Taken together, these results suggest that memory of the spatial structure and allocation of attention to the relevant parts of the scene are the dominating factors that lead to improvement in search performance.

### Guidance of movements from spatial memory

**Figure 6:**
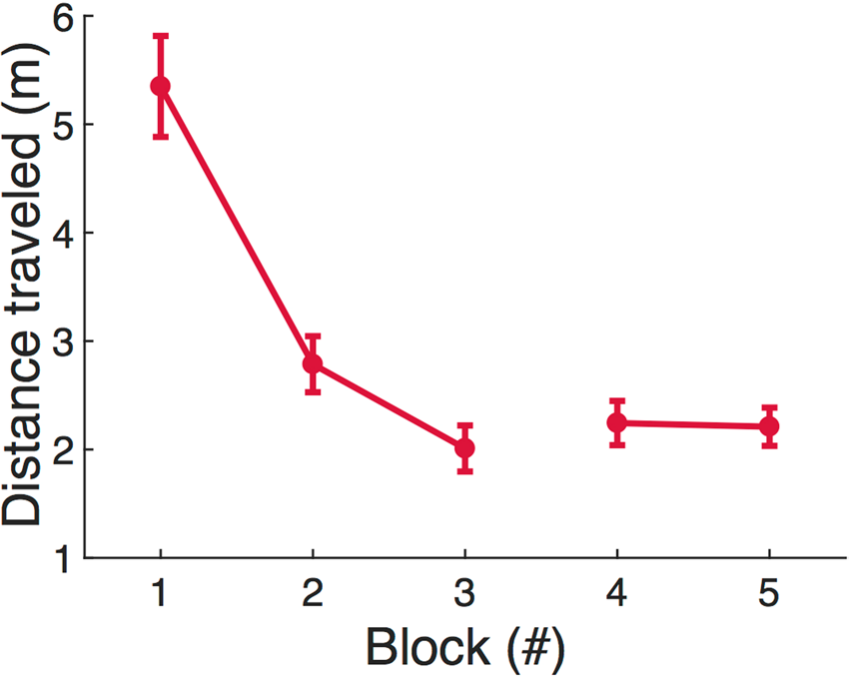
Total length of the trajectory, or distance traveled, from the beginning of the trial until target was found across blocks (N = 19). Data represent mean ± SEM.

**Figure 7:**
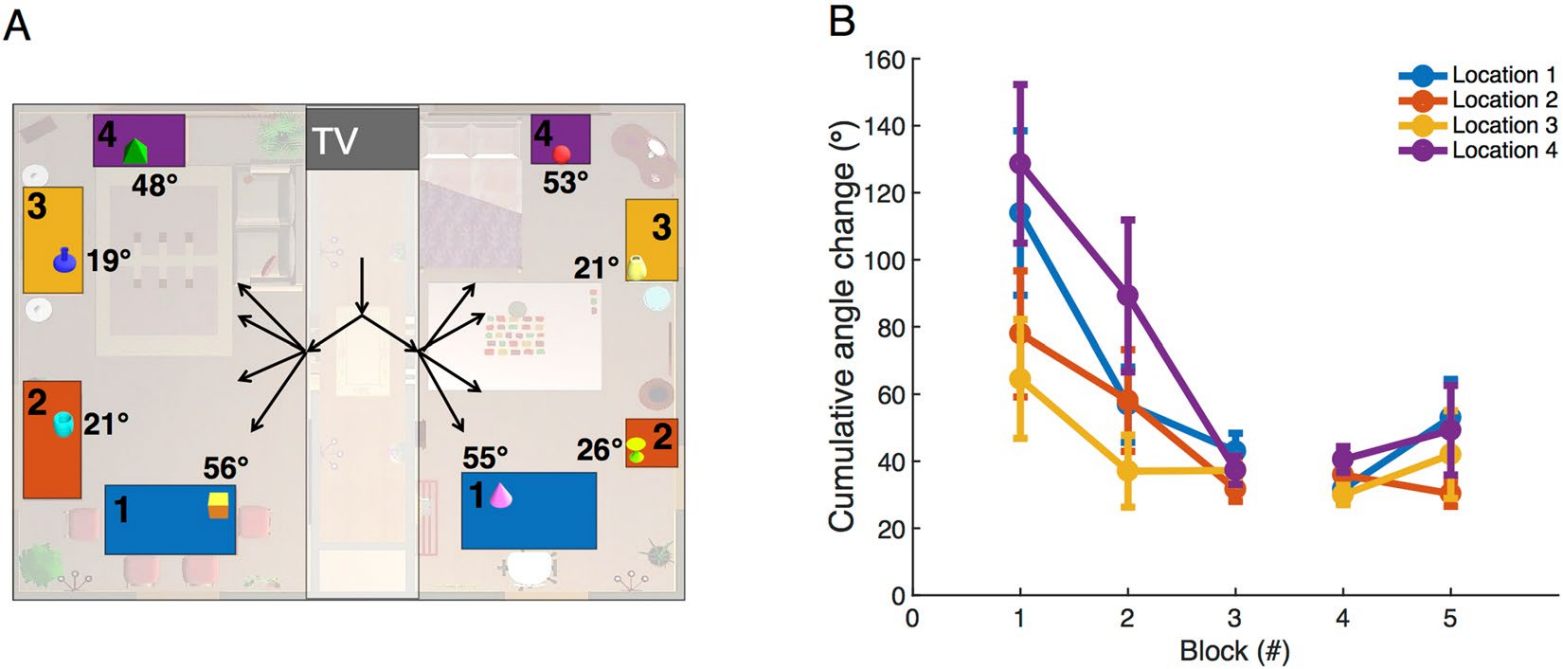
Analysis of head movements. (**A**) Top-down view of the room with targets shown on the surfaces. The angular deviations of the targets when looking straight ahead while entering the room in which they reside are marked next to the targets. The surfaces are colored by locations in the room: Locations 1–4. (**B**) Cumulative angular change in head direction from when the scene became visible until target was found (N = 19). Data represent mean ± SEM.

Given that subjects rapidly learn the structure of the space, it seems likely that this allows more efficient planning of body movements. To address this issue we examined the change in total distance traveled while searching as a function of trial block. This is plotted in Fig. 6. We summed the change in the coordinates of head position to calculate the total path distance in a trial. The distance traveled differed significantly across five blocks (*F*(4, 94) = 50.52, *p* = 0, *η*^2^ = 0.69) and the distance traveled in Block 2–5 was significantly lower than that of Block 1 (*p* = *0*). There was a significant increase in distance traveled from Block 3 to Block 4 when target locations changed (*p* = 0.045), but no significant difference was found between Block 4 and Block 5 (*p* = 0.45). We also calculated the cumulative change in head direction (relative to the angle at the start of the trial). Because the targets were in different locations in the rooms and required different magnitudes of head turning, we separated the data based on target location, as shown in Fig. 7A. The cumulative change in head direction (Fig. 7B) decreased over the first three blocks, but was different among locations (two-way ANOVA, block: *F*(2, 397) = 13.16, *p* < 0.001, partial *η*^2^ = 0.062; location: *F*(3, 397) = 3.32, *p* = 0.02, partial *η*^2^ = 0.024; interaction: *F*(6, 397) = 0.96, *p* = 0.45, partial *η*^2^ = 0.014). When the cumulative angles were compared between Block 1 and Block 3, significant reductions were found in Locations 1, 2 and 4 (paired *t*-test, L1: *t*(28) = 2.55, *p* = 0.009, Cohen’s *d* = 0.47; L2: *t*(28) = 2.26, *p* = 0.016, Cohen’s *d* = 0.42;, L4: *t*(29) = 3.24, *p* = 0.002, Cohen’s *d* = 0.59), but the difference was not significant for Location 3 (*t*(30) = 1.53, *p* = 0.068, Cohen’s *d* = 0.28). Therefore, changes in heading direction decreased with more experience. Changing target location in Block 4 did not have an effect.

**Figure 8:**
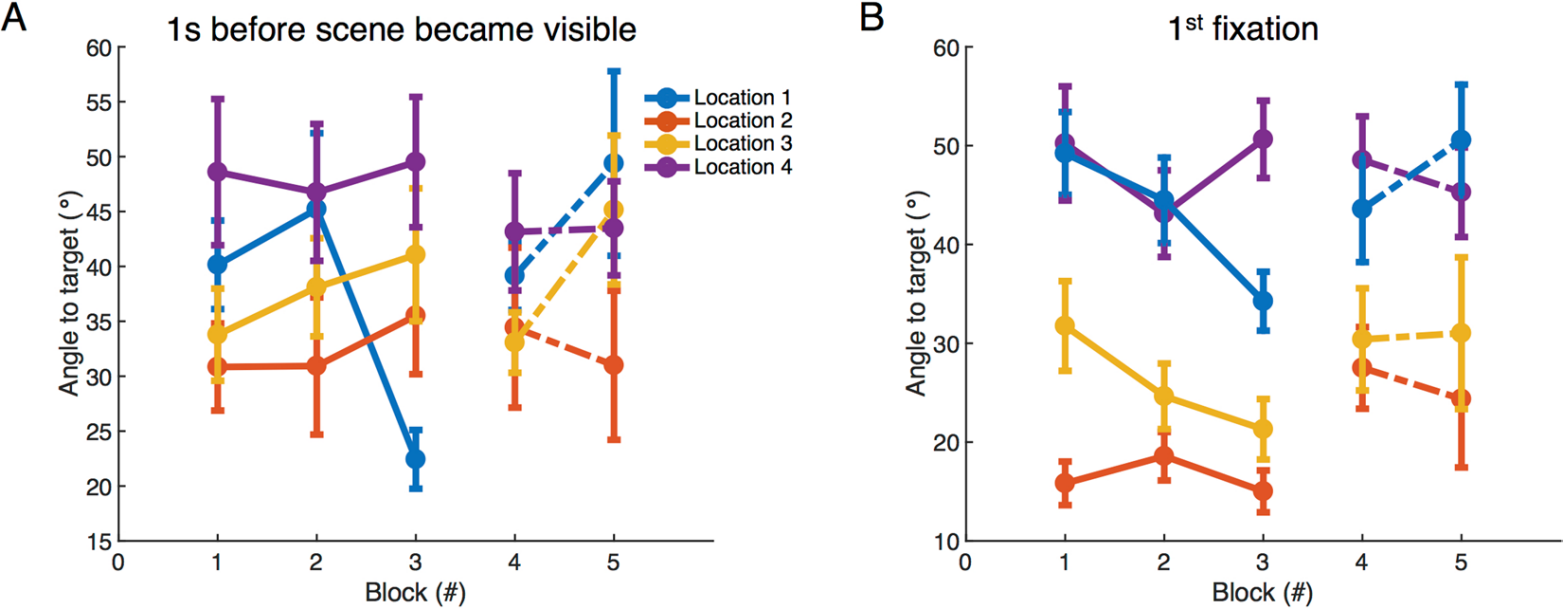
Orienting head to target before and after the inside of the room was visible. The four locations are based on those indicated in Fig. 7A. (**A**) Angles between head direction and target one second before the room is visible for Locations 1–4 across search blocks (N = 19). (**B**) Angles between head direction and target while making the first fixation in the room for Locations 1–4 across search blocks (N = 9). Data represent mean ± SEM. Although the figure shows between-subject error bars, the statistical analyses were based on within-subject comparisons.

To understand the extent to which subjects learnt to direct the head towards the targets, the angular difference between head direction and the direction from head to target was calculated and is plotted in Fig. 8 for each of the 4 target locations, as a function of Block. Note that for Block 4 the old target location was used as the reference. The angle between head direction and direction to target one second before the room was visible is shown in Fig. 8A, and the angle during first fixation in the room is shown in Fig. 8B. Note that the averages of angles in the thirty-frame window around 1 sec before the scene became visible and around the first fixation were used. In the first three blocks, the angle 1 sec before scene became visible was significantly different between four locations but not between blocks (two-way ANOVA: block: *F*(2, 191) = 0.31, *p* = 0.74, partial *η*^2^ = 0.003; location: *F*(3, 192) = 4.3, *p* = 0.005, partial *η*^2^ = 0.067; interaction: *F*(6, 192) = 1.6, *p* = 0.15, partial *η*^2^ = 0.051). The angle during first fixation was significantly different between blocks and also locations (two-way ANOVA: block: *F*(2, 215) = 3.01, *p* = 0.05, partial *η*^2^ = 0.029; location: *F*(3, 216) = 45.4, *p* = 0, partial *η*^2^ = 0.4; interaction: *F*(6, 216) = 1.6, *p* = 0.16, partial *η*^2^ = 0.044). In both cases, there is a significant reduction in angular error of head direction from the target between Blocks 1 and 3, but only for the target in Location 1 (1 sec prior: *t*(36) = 1.74, *p* = 0.045, Cohen’s *d* = 0.084; first fixation: *t*(17) = 2.95, *p* = 0.0045, Cohen’s *d* = 0.51). Location 1 required the smallest change in head angle in order to orient to the target when subjects walked down the hallway before entering the room, so that might underlie the effect for target at that location. Overall the head error data do not provide a good case that head error is reduced across trials possibly because the data is quite noisy, and the constraints of the head mounted display may have lead subjects to restrict head rotations. The question requires further investigation with a lighter HMD.

## Discussion

In this study we examined the nature of the memory representations that guide search in 3D immersive environments. When targets were moved to different locations on the fourth search episode, subjects fixated the old location of the target on 58% of trials. On 44% of trials the old location was one of the first two fixations. These saccades were presumably programmed on the basis of spatial memory, since the targets were easy to find and quite salient. Since subjects still made 20% errors in room selection on the third search episode, memory was not perfect, but search was still heavily weighted towards a spatial memory-based strategy. Surprisingly, however, general search performance was not disturbed much when locations were shuffled. Thus memory for structure of the space was sufficient for efficient search even when the specific spatial coordinates of the target were not known. When analyzing what was learned about the structure of the environment, we found that fixations were progressively restricted to relevant parts of the space. Consistent with the findings in Li *et al*.^25^, better choice of room was found as a result of experience. Over blocks the proportion of fixations to the correct side of the room, to relevant surfaces, and to the correct surface also increase. This finding is compatible with the idea that subjects learned the structure of the space at different spatial scales: room, room side, surfaces, and specific location. Subjects learned very quickly where not to look. Such a representation allows efficient guidance of eye movements, since subjects can restrict search to only some parts of the space. This is supported by the reduction in total path length and cumulative head rotation during search that we found. There was also some suggestion that subjects were orienting their heads towards the targets even before they were visible in the case of the two targets at the sides of the rooms, although this requires further investigation.

The improvement of search performance in the first three blocks, especially in the incorrect room, replicates our previous findings that suggest that spatial context is learned very quickly^25^. Draschkow & Võ also found similar learning of context in a real apartment, which appeared to result from incidental fixations^4^. The new manipulation of shuffling the locations in Block 4 barely affected performance. In the cases in which the target was moved to a different surface within the same room, subjects scanned mostly only the relevant surfaces, and were able to find the target as fast as in previous blocks, although they were likely to check the old location of the target as well and that might add a small cost. The primary cost was the greater probability of entering the incorrect room. In that case subjects could rapidly exclude the room by restricting gaze to the relevant surfaces (maximum of four). Within a few trials after targets were shuffled, subjects appeared to realize that locations of the targets changed every trial and thus adopted a strategy of searching for targets based mostly on their knowledge of the general layout of the room. In Block 5, targets returned to their original locations. Within a few trials, subjects appeared to realize this and directed their attention to the old location reliably so that performance was at the same level as in Block 2 and 3. Thus the search strategy again incorporated the use of memory for both the global structure and target locations. Manginelli and Pollman performed a similar manipulation in a 2D search paradigm with T’s and L’s^29^. Despite the difference in the paradigms and the overall learning rate, the results are quite similar, indicating a tendency to code targets by their location even when the context is hard to learn.

The search targets we used in the experiment were geometric objects that do not have obvious associations with their surrounding items. As shown by Võ and Wolfe^24,30^, in naturalistic images scene semantics often dominate search performance rather than episodic memory from search experience. Using geometric objects allowed us to assess the effect of episodic memory on search, without the effects of scene semantics. The choice of the correct room, the correct side of the room, the correct surface, and the correct location on the surface all suggest episodic memory of spatial location. However, there is evidence for some effect of scene semantics since subjects preferentially fixated surfaces in the early trials, indicating that a prior that small objects will be found on surfaces was used. Thus spatial learning in this kind of context could occur very rapidly since humans have presumably learned the basic statistical regularities of indoor environments. For example, in the present experiment subjects rarely look at the ground and ceilings for targets, demonstrating the existence of strong priors.

Previous work with 2D images has also provided evidence for memory representations at different spatial scales. Hollingworth^31,32^ showed that both local and global image structure learnt on previous trials affected search, and that disrupting spatial relationship impaired search performance. With simple stimuli, an effect of local context is only found after extensive experience^33–35^, although global aspects of the scene dominate the contextual cueing effect with real-world scene images^36^. With realistic immersive environments, we found that local contextual cues provided by neighboring objects had little effect^25^ and the current results indicate that global cues such as the correct room and correct surface are more important. Although the memory representation exists at different spatial scales, it does not need to be accessed in a coarse to fine manner. Indeed the restriction of fixations to surfaces developed quickly even though room choice was occasionally incorrect. Marchette, Ryan and Epstein^37^ found that memories at different spatial scales are to some extent independent, in conflict with Brooks *et al*.’s finding^37,38^. In our experiments, subjects appeared to learn the room, the side of the room, and the correct surface in the room at similar rates, and it is unclear whether memory representations at these different scales are organized hierarchically or independently.

The present results suggest that visual search strategies in realistic environments are strongly dependent on spatial memory. This most likely becomes an important factor when body movements are involved, because of the accompanying energetic costs. Recent approaches to understanding sensory-motor decisions reveal a critical role for rewards and costs in momentary action choices such as those involved in the search process here^39,40^. Although there is extensive evidence about the role of reward on oculomotor neural firing in neurophysiological paradigms^41^, it is unclear how this machinery controls natural behaviors. In addition, less is known about dopaminergic modulation of the neural circuitry underlying head and body movements at least at the cortical level. Our previous work compared visual search in immersive environments, with search in 2D images, where the head was fixed and only the eyes moved. We found that in 3D, subjects stayed in the incorrect room for a longer time initially and were better at learning the correct room, presumably because of the high cost of moving between rooms in 3D. These results, together with the present findings suggest that the costs of moving the head and the body are an important aspect of visually-guided behavior in natural environments, and must be considered for an integrated understanding of decision making in natural tasks. Although it is plausible to suggest that spatial memory representations are adopted in order to minimize energetic costs, the findings in our experiment are somewhat equivocal. While the total distance traveled dropped sharply with experience, it did not increase very much in Block 4, when the targets were moved to a new location. Thus it might be argued that subjects simply learned an efficient strategy for searching the space, unrelated to the memory of target location. After experience, subjects walked just inside the door and quickly scanned the surfaces and this strategy is effective even if the correct surface is not known. However, the adoption of the shorter travel paths depended on the spatial knowledge of the layout so there is clearly a tendency to reduce walking distance. Teasing apart these different explanations will require a paradigm where visually guided search is more difficult. Some aspect of gaze behavior are unaffected by moving around^42^, so exactly how costs affect search in ambulatory contexts requires further examination.

It seems clear that examination of scene memory needs to explore realistic environments where subjects move around. In a comprehensive review, Chrastil and Warren concluded that idiothetic information deriving from efferent motor commands and sensory re-afference aid the development of spatial memory^9^. They also concluded that the allocation of attention is a critical factor. This has been demonstrated by other experiments using real environments that do not explicitly investigate visual search. For example, Tatler & Tatler found that memory in a real environment was best when subjects were asked to attend to tea-related objects^43^. Drashkow & Võ found that object manipulation influenced memory^4^. Droll & Eckstein found that changes were easier to detect if the objects had previously been fixated^44^. In a paradigm similar to the one used here, where objects were changed following experience in virtual environments, Karacan & Hayhoe and Kit *et al*. found evidence that gaze was attracted to the changed region^26,45^. However, the present paradigm involved active search, so the results are difficult to compare. In summary, there are many complex factors underlying the development and structure of scene memories, many of which involve active body movements, and it appears that search in 2D displays reveals only a subset of those factors.

To summarize, humans typically conduct visual search in dynamic, large-scale environments requiring coordination of eye, head and body movements. Our results demonstrated that learning of the structure of the environment occurred rapidly, and attention was quickly narrowed down to relevant regions of the scene at multiple spatial scales. Memory of the large-scale spatial structure allows more energetically efficient movements, and this may be an important factor that shapes memory for large-scale environments.

## Methods

### Experimental Environment

The virtual environment was composed of a bedroom and a living room (3 m × 6 m each) connected by a hallway (1 m × 6 m) in between (Fig. 1A). It was generated in FloorPlan 3D V11 (IMSI) and rendered by Vizard 4 (WorldViz). Subjects saw the virtual environments through an nVisor SX111 head-mounted display (NVIS), which was fitted with a ViewPoint eye tracker (Arrington Research). The resolution of the HMD was 1,280 × 1,024 and the field of view (FOV) is 102° (in total, 76° each eye) horizontally and 64° vertically. Motion tracking was performed by HiBall motion tracking system (ThirdTech) at 600 Hz, and the latency for updating the display after head movement was 50 to 75 ms. The left eye was tracked at a sampling rate of 60 Hz. Tracker accuracy was about 1°. The calibration of the eye tracker was performed before, in the middle of, and at the end of the experiments on a nine-point (3 × 3) grid shown in the HMD. Recalibration was conducted when track loss or drift was observed, although we tried to minimize the amount of recalibration as that interrupts the task. Data with frequent track loss and poor calibration at the end of the experiment were excluded. At the end of the experiment, the synchronized videos of the scene display, eye tracking data, and metadata (head position, object position, and event timing) were saved in an MOV file for following data analysis and for verification of the automated analysis. Subjects clicked on a button on a Wiimote (Nintendo) when the target was found, but they were able to trigger the button press to end the trial only when they were within 1.5 m from the target and were looking at the target so that the target was within the central 70% of the screen. This prevented subjects from clicking the button randomly without locating the target. When the Wiimote button was triggered successfully, a ‘Trial Done’ message would show up on the screen to signal the completion of a trial.

#### Targets

Target objects were a set of eight different uniform-colored, geometric-shaped objects placed on eight different pieces of furniture, such as cabinet, dining table, desk, side tables and TV table, four in each room. The visual angle that targets subtended was approximately 2° to 2.5° on average when viewed from entry to the center of a room. Subjects usually found the target prior to reaching the center of the room. The same set of targets was searched for in five search blocks. Each target was searched for once in each block of trials, and thus there were eight trials in each block. The locations of the targets were the same during the first three blocks. In Block 4, the targets were moved to a different surface randomly chosen from all the other locations that contained target objects in the first three blocks. That is, the target locations were shuffled. This was true for each trial in the fourth block. Targets could move to a different surface in the same room or in another room. In Block 5, the targets returned to their original locations, where they were in the first three blocks. Subjects were not told that the targets shuffled in Block 4 or that they moved back to the old locations in Block 5.

#### Procedure

Prior to the start of the experiment, the eye tracker was calibrated and then subjects were given instructions about the procedure while standing in front of the TV on the hallway within the VR environment. Then they were instructed to proceed from the hallway to one of the two rooms for one minute to become familiar with navigating in a virtual environment and looking around freely. Following free exploration the subjects returned to the hallway to start the main task: visual search for targets for five blocks, for a total of 40 trials. Each trial followed the same structure: at the beginning of a trial, subjects returned to the hallway from any room they visited and approached the TV screen at the end of the hallway, on which an image of the next target object would show up. Then subjects had to turn around and decide which room to enter to look for the target. They were allowed to freely traverse between the two rooms until the target was located, which usually took one to three room visits. Each room had a door that opened when approached but blocked view of the target until the room had been entered. The order of the target objects was randomized within each search block and across subjects. The targets on successive trials were never repeated.

#### Gaze analysis

An in-house program written in Vizard was used to reconstruct the environment from the metadata and generate a data file that included the positions of head, eye, objects and identity of the object that the gaze intersected at each frame. The eye tracking data were then analyzed in Matlab. A median filter was first applied to remove outliers, and then a three frame moving average filter smoothed the signals. Next, the data were segmented into fixations and saccades using a velocity-based algorithm. A fixation was detected when the velocity of the signal was lower than 60 degrees/s and lasted at least for 100 ms. Note that a relatively high velocity threshold was used due to the signal from the low-velocity vestibular-ocular reflex, which adds to the velocity of the eye movement signal while head is moving. Transient track losses were ignored if the fixation was made to the same object prior and after the loss. When consecutive fixations were less than 80 ms and 1.5° apart, they were combined as one fixation. The labeling of the object being fixated was determined by the program with a window of 80 × 80 pixels that spans approximately 5° × 5° of visual angle centered at the gaze point on each frame. This enables the mapping of the 2D gaze coordinates for each frame in the data. A relatively large window was used to allow for possible drifts of the eye signal and inaccuracy of the calibration. Another reason worth noting was that the target was easily visible even without direct fixation so subjects may not have executed corrective saccades for precise fixation on the targets. Next, the eye movement data were segmented into trials. We defined the start of each trial in our later analysis as the point in time when the first room entry was made. The end of the trial was the time when the target was found, which was defined as the time when the subjects made the first fixation to the target without making subsequent fixations to other objects before the Wiimote button was clicked. This eliminated cases where subjects were unaware that they had fixated the target and fixated other objects before returning to the target. For analyzing fixations to surfaces, another analysis was performed in which a box region around each relevant surface, 40 cm above and 20 cm below, was defined. Fixations that fell within these boxes were identified as fixations to the surfaces. Again the large size of the boxes was used to tolerate for drifts in the eye signal.

#### Participants

Twenty-one students from the University of Texas at Austin participated in the experiment, which was approved by the University of Texas Institutional Review Board (IRB: 2006–06–0085) and were performed in accordance with relevant guidelines and regulations. All of the subjects had normal vision and provided informed consent to participate. Class credit or monetary compensation was provided. Of all the subjects, two skipped the first trial and were excluded from all analysis; and ten subjects did not have reliable eye tracking data that allowed parsing fixations, so were excluded from all the data analysis related to fixations, including search time (since the end of the trial was defined by the first fixation to the target), fixations in general and fixations to surfaces. Track loss is a common issue in the HMD and eye tracker combination we used in this experiment, primarily because of the awkward geometry where the eye tracker camera must fit in between the helmet and the eye. In more recent helmets the eye tracker optics are embedded within the HMD optics, allowing for much easier and more reliable tracks. The heavy weight and tethering cables also make the alignment precarious and can lead to track losses during the session if the helmet moves on the head. Thus only nine subjects were included in those analyses. For other analyses, such as analysis of percentage of correct room choices and analyses of head movements that were not related to fixations, 19 subjects with full behavioral data (no trials skipped) were included.

#### Data analysis

All analyses of the data extracted from the reconstruction were performed in Matlab. Search time (time spent to find the targets) and number of search fixations were calculated and used as the indicators of search performance. Time and fixation counts in the correct room (the room that contained the target on that trial) and the incorrect room were calculated. Inside the rooms, number of fixations to the surfaces and non-surface areas were computed. For head movement analysis, the angles between head direction and direction from the participant to the target in the thirty-frame window around 1 sec before the room became visible and around first fixation were calculated separately. The cumulative angular change in head direction was also calculated. Data that were three standard deviations away from the mean were excluded. An alpha level of 0.05 was used for all statistical tests. Levene’s test of homogeneity was used to test the equality of variances between groups. When variances were equal, standard ANOVA was used to test differences of means, and Tukey’s HSD test was used for post-hoc analysis. When the assumption of homogeneity in variances was violated, Welch’s test was used to test differences in means and Games-Howell post-hoc test was used instead.

#### Data availability

The datasets generated collected and analyzed during the current study are available from the corresponding author on reasonable request.
